# A Novel Method for the Accurate Evaluation of Poisson's Ratio of Soft Polymer Materials

**DOI:** 10.1155/2013/930798

**Published:** 2013-04-23

**Authors:** Jae-Hoon Lee, Sang-Soo Lee, Jun-Dong Chang, Mark S. Thompson, Dong-Joong Kang, Sungchan Park, Seonghun Park

**Affiliations:** ^1^System Research and Development, Samsung Heavy Industries, Geoje 656-710, Republic of Korea; ^2^School of Mechanical Engineering, Pusan National University, Busan 609-735, Republic of Korea; ^3^Institute for Skeletal Aging & Orthopedic Surgery, College of Medicine, Hallym University, Chuncheon 200-704, Republic of Korea; ^4^Department of Engineering Science, University of Oxford, Oxford OX1 3PJ, UK; ^5^Department of Urology, College of Medicine, University of Ulsan, Ulsan 682-714, Republic of Korea

## Abstract

A new method with a simple algorithm was developed to accurately measure Poisson's ratio of soft materials such as polyvinyl alcohol hydrogel (PVA-H) with a custom experimental apparatus consisting of a tension device, a micro X-Y stage, an optical microscope, and a charge-coupled device camera. In the proposed method, the initial positions of the four vertices of an arbitrarily selected quadrilateral from the sample surface were first measured to generate a 2D 1st-order 4-node quadrilateral element for finite element numerical analysis. Next, minimum and maximum principal strains were calculated from differences between the initial and deformed shapes of the quadrilateral under tension. Finally, Poisson's ratio of PVA-H was determined by the ratio of minimum principal strain to maximum principal strain. This novel method has an advantage in the accurate evaluation of Poisson's ratio despite misalignment between specimens and experimental devices. In this study, Poisson's ratio of PVA-H was 0.44 ± 0.025 (*n* = 6) for 2.6–47.0% elongations with a tendency to decrease with increasing elongation. The current evaluation method of Poisson's ratio with a simple measurement system can be employed to a real-time automated vision-tracking system which is used to accurately evaluate the material properties of various soft materials.

## 1. Introduction

Recently, polyvinyl alcohol hydrogel (PVA-H) has attracted attention due to its importance in the use of an artificial load-bearing material for human articular cartilage [1–4]. Because of the excellent mechanical strength and bioaffinity of PVA-H [2, 5–8]; diverse manufacturing methods of PVA-H have been investigated to improve its mechanical properties, and the suitability of PVA-H as a cartilage replacement material has been examined [3, 9–13]. However, numerical analysis of the *in vivo *mechanical behavior and clinical applicability of PVA-H has been popular with various analytical models due to difficulties in experimental measurements [14–16]. In order to use those analytical models, the material properties of PVA-H, such as Young's modulus and Poisson's ratio, should be accurately provided; for example, the use of incorrect Poisson's ratio values induces misleading analytical results of shear stress at the contacting interface of articular cartilage [16]. However, the constant values of Young's modulus and Poisson's ratio could not be defined for gel type soft materials such as PVA-H, because these materials are known to have time-dependent nonlinear or hyperelastic material properties with large deformation under a small applied load [17, 18]. 

Poisson's ratios of metallic materials are generally measured using strain gauges that are directly attached to the metal surface with an appropriate orientation. In contrast, the hyperelastic or nonlinear nature of soft materials makes it difficult to attach strain gauges to the material surface. Therefore, in previous studies, Poisson's ratio of PVA-H was determined by using specially designed high-precision experimental equipments with a very expensive high-resolution charge-coupled device (CCD) and a complementary metal-oxide semiconductor (CMOS) camera [19–23]. Although these high-precision devices are adopted to optically measure the PVA-H Poisson's ratio, a persistent misalignment problem between samples and loading devices as well as between optical and loading devices still exists. This problem directly affects the accuracy of Poisson's ratio measurements of PVA-H.

In the present study, a novel method with a simple algorithm is proposed to accurately measure Poisson's ratio of soft and highly deformable materials such as PVA-H, although there is misalignment between measurement samples and experimental devices. 

## 2. Materials and Methods

### 2.1. Sample Preparation

An amount of 20 wt% of PVA powder (341584-poly(vinyl alcohol), Sigma-Aldrich, MO, USA), which results in 80 wt% of water content correspondent to human articular cartilage, was dissolved in phosphate-buffered saline (P5493, Sigma-Aldrich, MO, USA). The PVA solution was boiled and stirred at 120°C for 1 h using a stirring machine (SP131320-33, Thermo Scientific, MA, USA). Then, the boiled PVA solution was cast into a sheet with a thickness of 3 mm by using a custom mold. The mold with PVA-H was frozen at –20°C for 10 h and thawed at 4°C for 20 h. This freezing-thawing cycle was repeated four times to enhance the mechanical properties of PVA-H, because the number of freezing-thawing cycles was reported to affect the material structures and mechanical properties of PVA-H [24–27]. Finally, PVA-H specimens with a length, a width, and a thickness of 50, 10, and 3 mm, respectively, were prepared.

### 2.2. Measurements of PVA-H Deformation under Tension

**Figure 1:**
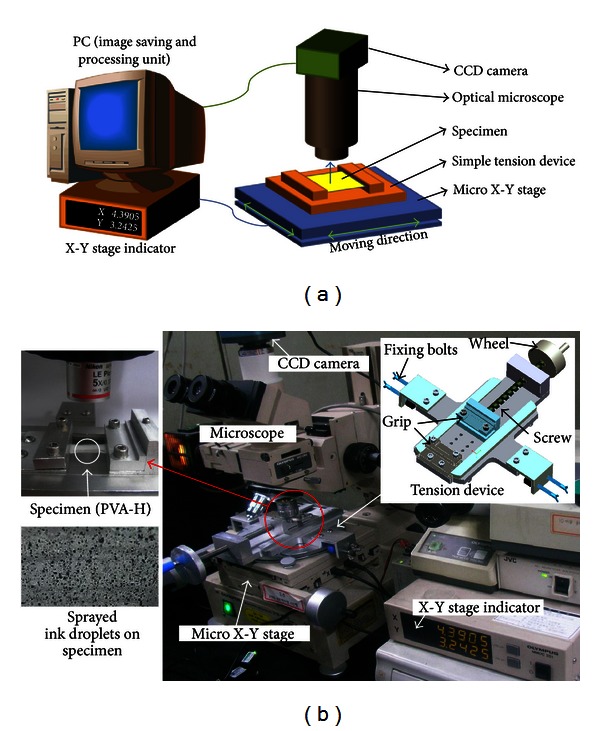
Experimental apparatus—(a) schematic of a PVA-H loading and deformation measurement device and (b) experimental setup.

An experimental apparatus to measure initial and final PVA-H deformation under tension consists of three components: a custom tension device to apply tension to PVA-H with simple operating mechanism, a custom micro X-Y stage to place the tension device in a desired position, and an optical microscope (Olympus Measuring Microscope STM, Olympus, Tokyo, Japan) and a CMOS camera (iCM 5.0, IMT i-Solution, BC, Canada) to acquire the quadrilateral image of PVA-H (Figure 1). 

Prior to experiments, ink droplets were sprayed on the sample surface as marker points to measure the amount of sample deformation under tension. To make uniformly distributed ink spots on the sample surface, moisture on the surface was first properly eliminated by using a paper towel, and then a black enamel ink was sprayed from a distance. Each ink spot could be distinguished by a thin coating when imaged from the optical microscope. Then, the PVA-H sample was fixed on both grips of the tension device and stretched by rotating a wheel and screw. No sensor was used to measure applied loads because strain measurements are the main purpose of this study. 

**Figure 2:**
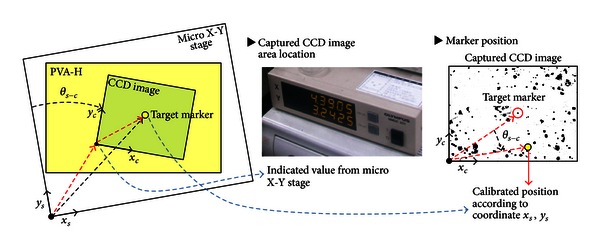
Global coordinates of a target position on the PVA-H surface.

In order to evaluate Poisson's ratio of PVA-H, the global positions of four marker points on the PVA-H surface need to be first measured before and after deformation.  Figure 2 shows the process used to calculate the global coordinates of an example target marker, where the  *x*_*s*_-*y*_*s*_ coordinate system represents a moving coordinate system to indicate the movement of a micro X-Y stage and the *x*_*c*_-*y*_*c*_ coordinate system indicates the location of the target markers in the captured CCD image. Because the global positions of the four selected markers were obtained by combining the two different coordinate systems (i.e., one on the micro X-Y stage, and the other on the CCD image), misalignment between the two coordinates should be adjusted. For this adjustment, the position data of the CCD image were rotated by an angle, *θ*_*s*−*c*_, that is equal to a difference between the two coordinates, thus generating the calibrated global position (Figure 2). The angle *θ*_*s*−*c*_ was measured by moving the micro X-Y stage to either side of the *x*_*s*_- or *y*_*s*_-axis. These global coordinates of the four arbitrary target markers were used as the vertices of a quadrilateral element in finite element analysis (FEA).

### 2.3. Poisson's Ratio Calculation Method

**Figure 3:**
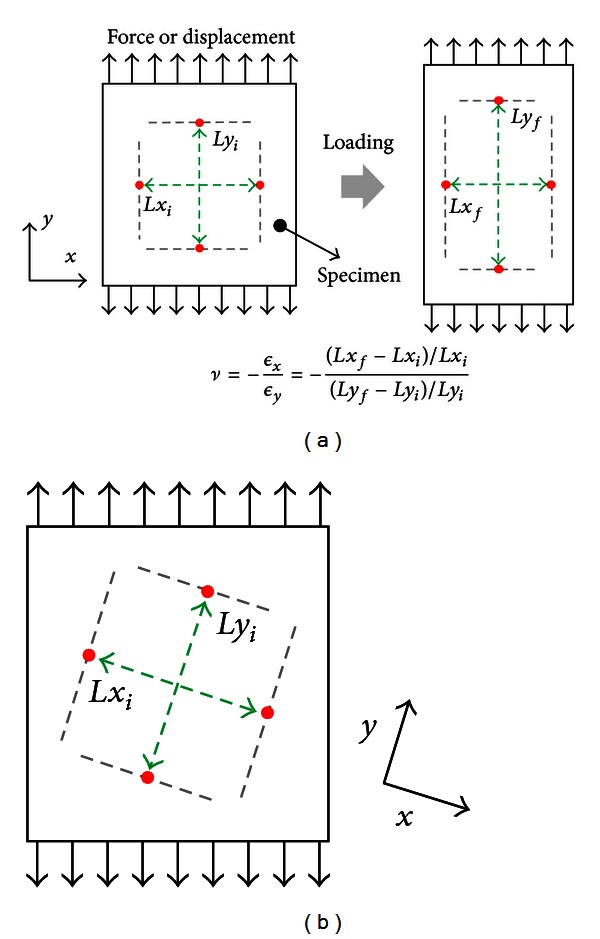
Schematic of the standard method of calculating Poisson's ratio—(a) Poisson's ratio calculation on a 2D plane and (b) misalignment between coordinates and loading axes.

When a PVA-H specimen is stretched in the axial direction, its width and thicknesses in the other two transverse directions decrease. Poisson's ratio is defined as the ratio of transverse strain to the axial strain.  Figure 3(a) shows a schematic of a generally used method for calculating Poisson's ratio. Here, the *y*-axis represents the axial direction, and *ϵ*_*x*_ and *ϵ*_*y*_ are the transverse and axial strains, respectively. In this generally used previous method, Poisson's ratio (*ν*) was calculated from the initial (*x*_*i*_, *y*_*i*_) and final (*x*_*f*_, *y*_*f*_) coordinates of the four arbitrary marker points measured on the sample surface before and after deformation by using the following equation:

(1) $$\begin{array}{c} x_{f}=x_{i}\left(1-\epsilon_{y}\nu \right),\;\;y_{f}=y_{i}\left(1+\epsilon_{y}\right). \end{array}$$

**Figure 4:**
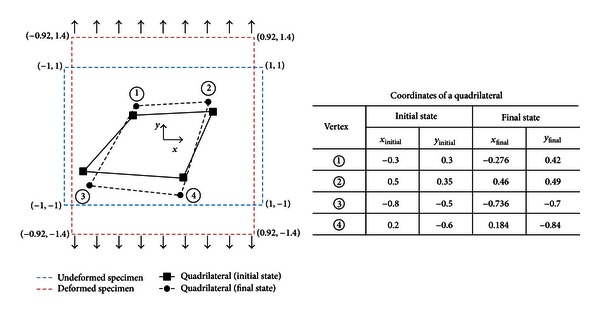
Coordinates of the four marker points before and after deformation.

For example, by assuming a PVA-H specimen to be perfectly aligned, rectangular, isotropic, and homogeneous, the calculated Poisson's ratio is 0.2 for a transverse strain of –0.08 and an axial strain of 0.4. However, as shown in Figure 3(b), difficulties in perfectly aligning the sample, the optical microscope, and the loading device simultaneously during loading always produce misalignments of samples with respect to the loading direction as well as misalignments between measurement systems and loading devices. These misalignments result in inaccurate Poisson's ratio measurements, as shown in Figure 4; Figure 4 shows differences between the initial and deformed shapes of the rectangular specimen and changes in the coordinates of four marker points between before and after deformation when a sample is misaligned. Thus, (1) cannot be used to calculate Poisson's ratio for misaligned samples because the loading direction is not the principal direction of sample deformation. 

**Figure 5:**
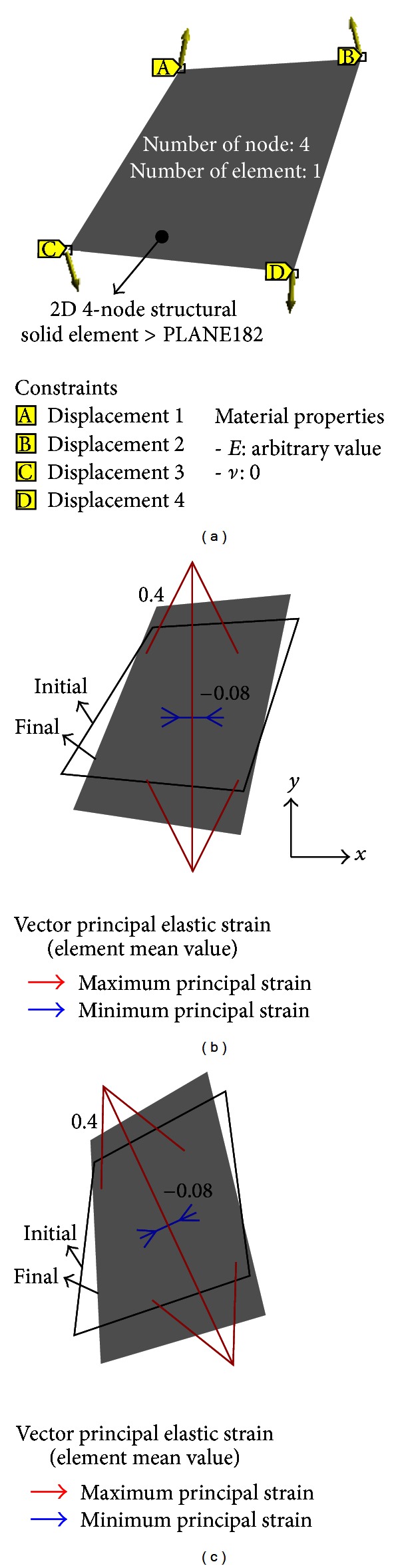
Finite element model and results: (a) FE model with applied conditions, (b) and (c) analysis results for the model in Figure 2 and the rotated model by 25°, respectively.

The present study proposes to calculate Poisson's ratio for misaligned samples by calculating the principal strains from the global positions of four arbitrarily chosen markers before and after deformation. In order to calculate the principal strains, the finite element method (FEM) was employed. Since the FEM involves the use of shape functions and strain formulations [28], it is convenient to identify changes in strain corresponding to changes in specimen geometry. An FE model constructed for the evaluation of Poisson's ratio is shown in Figure 5. The element type of a quadrilateral is PLANE182 in commercial FEM software (V13, ANSYS, PA, USA), which is a 2D solid element and a 1st-order element with four nodes per element, and therefore suitable for expressing the four vertices of a quadrilateral. Differences between the initial and final deformations were used as displacement constraints at the four vertices. 

**Figure 6:**
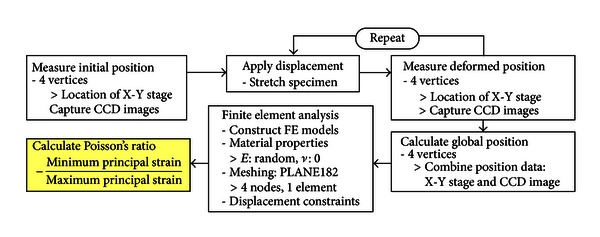
Procedure of Poisson's ratio evaluation.

Although Young's modulus should be generally provided to perform FE analysis, it was not needed in the current study because we were only focused on evaluating Poisson's ratio which could be obtained by calculating strains from changes in specimen geometry according to the displacement constraints of the quadrilateral. Thus, Young's modulus was given as a random value. Poisson's ratio is another important value that should be provided before FE analysis and initially assumed to be 0 to prevent strains in different directions from interacting with each other. Then, the final Poisson's ratio value of PVA-H material was determined from the ratio of the two principal strains acquired from FE analysis, which is the aim of the current study. The procedure for obtaining Poisson's ratio is briefly described in Figure 6, where the “Repeat” in the procedure implies the repeated calculations of Poisson's ratio at various elongations of a specimen.

To verify our proposed method, we used two different and simple FE models. The first model is shown in Figure 4 and the second is generated by rotating the first model by 25° in the counterclockwise direction. FE analysis produced the same principal strain values for both models; the maximum (*ϵ*_1_) and minimum (*ϵ*_2_) principal strains were 0.4 and –0.08, respectively, as shown in Figure 5. Therefore, the calculated Poisson's ratio by the formula *ν* = −*ϵ*_2_/*ϵ*_1_ was 0.2. The same Poisson's ratio value in the two different models validates that Poisson's ratio can be successfully calculated by four arbitrarily chosen marker points of the sample surface, although samples and experimental devices are misaligned. 

## 3. Results

**Figure 7:**
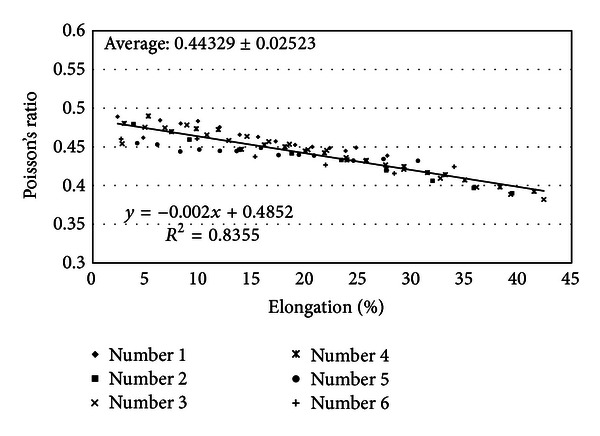
Poisson's ratio of the PVA-H as a function of elongation (different samples are denoted by different symbols in the legend).

Poisson's ratio of the PVA-H was calculated for a total of 6 specimens by manually incrementing the elongation in a stepwise manner. Poisson's ratio of the PVA-H calculated using the proposed method in this study ranged from 0.490 to 0.382 at 2.6–47.0% elongation and showed a tendency to decrease with increasing elongation (Figure 7). The average Poisson's ratio value from all 81 measurements was 0.44 ± 0.025, close to the value (~0.5) of incompressible materials such as rubber. For these Poisson's ratio measurements, both misalignments between the sample and loading device as well as between the optical and loading devices were intentionally made to prove our proposed method. Although the misalignment between the sample and the loading device was 32° for the 3rd specimen (symbol “×” in Figure 7), the resulting Poisson's ratio values were similar to those of the other specimens. This result validates that the persistent misalignment problem to prevent accurate Poisson's ratio measurements can be overcome by our proposed method.

One limitation of this experiment was that the experimental sequence of Poisson's ratio measurements was always in the increasing order of elongation. This limitation may cause a decrease in the Poisson's ratio values obtained at higher elongations because the greater time (i.e., approximately 30–50 min depending on samples) required to conduct experiments at higher elongations can cause a decrease in the water content of the PVA-H and thus a decrease in the Poisson's ratio; PVA-H has a porous structure with water, and changes in the water content possibly lead to changes in the hydrogel's mechanical properties (e.g., reduced incompressibility with decreasing water content). A further study is needed to determine whether lower Poisson's ratio values at higher elongations are due to the intrinsic material property of PVA-H or changes in the water content.

## 4. Conclusions

Poisson's ratio of PVA-H was successfully evaluated by a new method proposed in this study by using a simple custom experimental device. Poisson's ratio values obtained from the proposed method were in the range of 0.4–0.5 at approximately 2–50% elongations of PVA-H. The currently proposed method also allows the accurate measurements of Poisson's ratios of other soft materials and can be applied to a real-time automated vision-tracking system, although there are misalignments between samples and experimental devices.
